# Pinpointing the precise stimulation targets for brain rehabilitation in early-stage Parkinson’s disease

**DOI:** 10.1186/s12868-023-00791-7

**Published:** 2023-03-29

**Authors:** Hanna Lu, Jing Li, Li Zhang, Lin Meng, Yuping Ning, Tianzi Jiang

**Affiliations:** 1 G27, Multi-Centre, Department of Psychiatry, The Chinese University of Hong Kong, Tai Po Hospital, Hong Kong SAR, China; 2 Centre for Neuromodulation and Rehabilitation, The Affiliated Brain Hospital of Guangzhou Medical University, Guangzhou, China; 3 The Affiliated Brain Hospital of Guangzhou Medical University, Guangzhou, China; 4 Department of Mechanical and Automation Engineering, The Chinese University of Hong Kong, Hong Kong SAR, China; 5 Academy of Medical Engineering and Translational Medicine, Tianjin University, Tianjin, China; 6 The First School of Clinical Medicine, Southern Medical University, Guangzhou, China; 7 Brainnetome Center, Institute of Automation, Chinese Academy of Sciences, Beijing, China; 8 National Laboratory of Pattern Recognition, Institute of Automation, Chinese Academy of Sciences, Beijing, China; 9 Research Center for Augmented Intelligence, Zhejiang Lab, Hangzhou, 311100 China

**Keywords:** Parkinson’s disease, Transcranial magnetic stimulation, Scalp-to-cortex distance, DLPFC, Head model, Simulation

## Abstract

**Background:**

Transcranial magnetic stimulation (TMS) is increasingly used as a promising non-pharmacological treatment for Parkinson’s disease (PD). Scalp-to-cortex distance (SCD), as a key technical parameter of TMS, plays a critical role in determining the locations of treatment targets and corresponding dosage. Due to the discrepancies in TMS protocols, the optimal targets and head models have yet to be established in PD patients.

**Objective:**

To investigate the SCDs of the most popular used targets in left dorsolateral prefrontal cortex (DLPFC) and quantify its impact on the TMS-induced electric fields (E-fields) in early-stage PD patients.

**Methods:**

Structural magnetic resonance imaging scans from PD patients (n = 47) and normal controls (n = 36) were drawn from the NEUROCON and Tao Wu datasets. SCD of left DLPFC was measured by Euclidean Distance in TMS Navigation system. The intensity and focality of SCD-dependent E-fields were examined and quantified using Finite Element Method.

**Results:**

Early-stage PD patients showed an increased SCDs, higher variances in the SCDs and SCD-dependent E-fields across the seven targets of left DLPFC than normal controls. The stimulation targets located on gyral crown had more focal and homogeneous E-fields. The SCD of left DLPFC had a better performance in differentiating early-stage PD patients than global cognition and other brain measures.

**Conclusion:**

SCD and SCD-dependent E-fields could determine the optimal TMS treatment targets and may also be used as a novel marker to differentiate early-stage PD patients. Our findings have important implications for developing optimal TMS protocols and personalized dosimetry in real-world clinical practice.

**Supplementary Information:**

The online version contains supplementary material available at 10.1186/s12868-023-00791-7.

## Introduction

The rapid ageing of populations around the world poses an unprecedented set of challenges, the most significant of which is the rise in the prevalence of age-related neurodegenerative diseases, including Alzheimer’s disease (AD) and Parkinson’s disease (PD) [1]. Notably, the incidence rate of PD is increasing more quickly than that of AD [2]. The Global Burden of Disease Study predicts that the number of PD patients will double from around 7 million in 2015 to about 13 million in 2040 [3]. Aside from the growing number of patients, PD is a complicated, progressive neurodegenerative disease with a wide spectrum of premotor, motor and non-motor symptoms at different stages of the disease, but there are still very few evidence-based therapeutic methods for managing the depressive and cognitive symptoms in early-stage PD patients [1]. Even though pharmacological therapies have much helped to manage the motor symptoms [4], the negative consequences from dopamine administration to brain regions outside of the basal ganglia might cause or exacerbate cognitive problems in PD patients [5, 6].

Based on well-established PD models, dopamine projections are predominantly directed to basal ganglia and prefrontal cortex (PFC) [7], therefore, the neuroanatomical targets for brain stimulation are determined by the computational models of brain features. Transcranial magnetic stimulation (TMS), a non-invasive modality of brain stimulation in addition to deep brain stimulation (DBS), has gained significant interests as a safe and effective treatment for the main types of age-related neurodegenerative diseases, including AD and PD [8, 9]. The administration of TMS treatment over left dorsolateral prefrontal cortex (DLPFC) has shown encouraging results to enhance the cognition and motor functions in PD patients [10–12]. However, these studies have revealed a variety of findings [13]. The long-standing concerns regarding the heterogeneity derived from the clinical trials continue to be fiercely discussed recently [14]. The existing computational models of TMS are developed using the template from a general population, which may not closely resemble the head size and brain features in senior adults or PD patients. Moreover, scalp-to-cortex distance (SCD), as a key technical parameter of TMS, demonstrating the geometric distance from the scalp (i.e., TMS coil) to the cortex (i.e., cortical surface), has a significant detrimental impact on the electric fields (E-fields) induced by TMS [15]. While developing optimal TMS protocols is certainly important and therapeutically significant, the major challenges associated with the potential heterogeneity impede the personalized TMS treatment for the people living with early stage PD. The heterogeneity caused by brain features may be decomposed into two levels: (1) Inter-individual: differences in brain size and cortical features among early-stage PD patients; (2) Intra-individual: differences in treatment targets locations and corresponding variations in the SCDs of these targets.

Collectively, the longstanding absence of optimal treatment targets has resulted in larger differences between clinicians regarding the TMS used in the treatment of PD patients. With respect to the highly folded anatomical target (i.e., DLPFC), a rigorous quantitative approach to morphometric analysis is required to better quantify the cortical features in the context of brain atrophy at individual level. Thus, the main aim of current study was to systematically investigate the SCDs of the most popular used targets of left DLPFC and determine the optimal treatment targets in early-stage PD patients and age-matched normal controls. A second aim was to examine and quantify the SCD-dependent matrices of the TMS-induced E-fields using Finite Element Method (FEM).

## Materials and methods

### Participants

T1-weighted structural magnetic resonance imaging (MRI) scans were aggregated from two publicly accessible datasets [16]: (1) The NEUROCON study included 27 PD patients and 16 age-matched normal controls without a history of psychiatric or neurological diseases. (2) The Tao Wu dataset included 20 PD patients and 20 age-matched normal controls. According to the clinical standards of the Queen Square Brain Bank (QSBB) and the European Federation of Neurological Societies / Movement Disorder Society - European Section (EFNS/MDS-ES), all patients were in the early or moderate stage of PD (Hoehn and Yahr stages 1 to 2.5). We recruited 83 participants in total, comprising 47 early-stage PD patients and 36 age-matched normal controls.

In accordance with the ethical standards of the 1964 Declaration of Helsinki and its later amendments, the NEUROCON study has been approved by the University Emergency Hospital Bucharest ethics committee. Each patient signed a written informed consent form to participate in the study. Additionally, the study sites specifically secured consent for public sharing of the anonymized data. The demographics of the participants, in terms of age, sex, and the years of education, and the scores of cognitive functions were directly obtained from the NEUROCON and Tao Wu datasets. The current study was approved by the Clinical Research Ethics Committee of The Chinese University of Hong Kong (CUHK) and New Territories East Cluster (NTEC) (The Joint CUHK-NTEC).

### MRI acquisition

Details about the protocol of MRI acquisition can be reviewed on the official webpage of Parkinson’s Disease Datasets (http://fcon_1000.projects.nitrc.org/indi/retro/parkinsons.html). The structural MRI scans derived from the NEUROCON study were acquired on a 1.5T Siemens Avanto scanner with a thermo-plastic face mask to minimize head movements [16]. To better co-register to standard Montreal Neurological Institute (MNI) space, T1-weighted MRI scans were obtained for all participants using a magnetization-prepared rapid gradient-echo (MPRAGE) sequence (IR method, TR = 1940 ms, TE = 3.08 ms, inversion time (IT) = 1100 ms, voxel size 0.97 × 0.97 × 1 mm). The structural MRI scans from the Tao Wu database was acquired on a 3.0T Siemens Magnetom scanner. MPRAGE scans were obtained (TR = 1100 ms, TE = 3.39 ms, voxel size 1 × 1 × 1 mm) for the registration to the MNI space.

### Clinical and motor assessments

The modified Hoehn and Yahr (HY) Scale was used to evaluate the clinical and motor symptoms and disease progression in PD patients. The HY scale was originally described in 1967 and included five stages to PD. It has since been modified with the addition of stages 1.5 and 2.5 to account for the intermediate course of PD [17]. Mini-Mental State Examination (MMSE) was used to evaluate the global cognitive function [18].

### Surface-based morphometry analysis

**Fig. 1:**
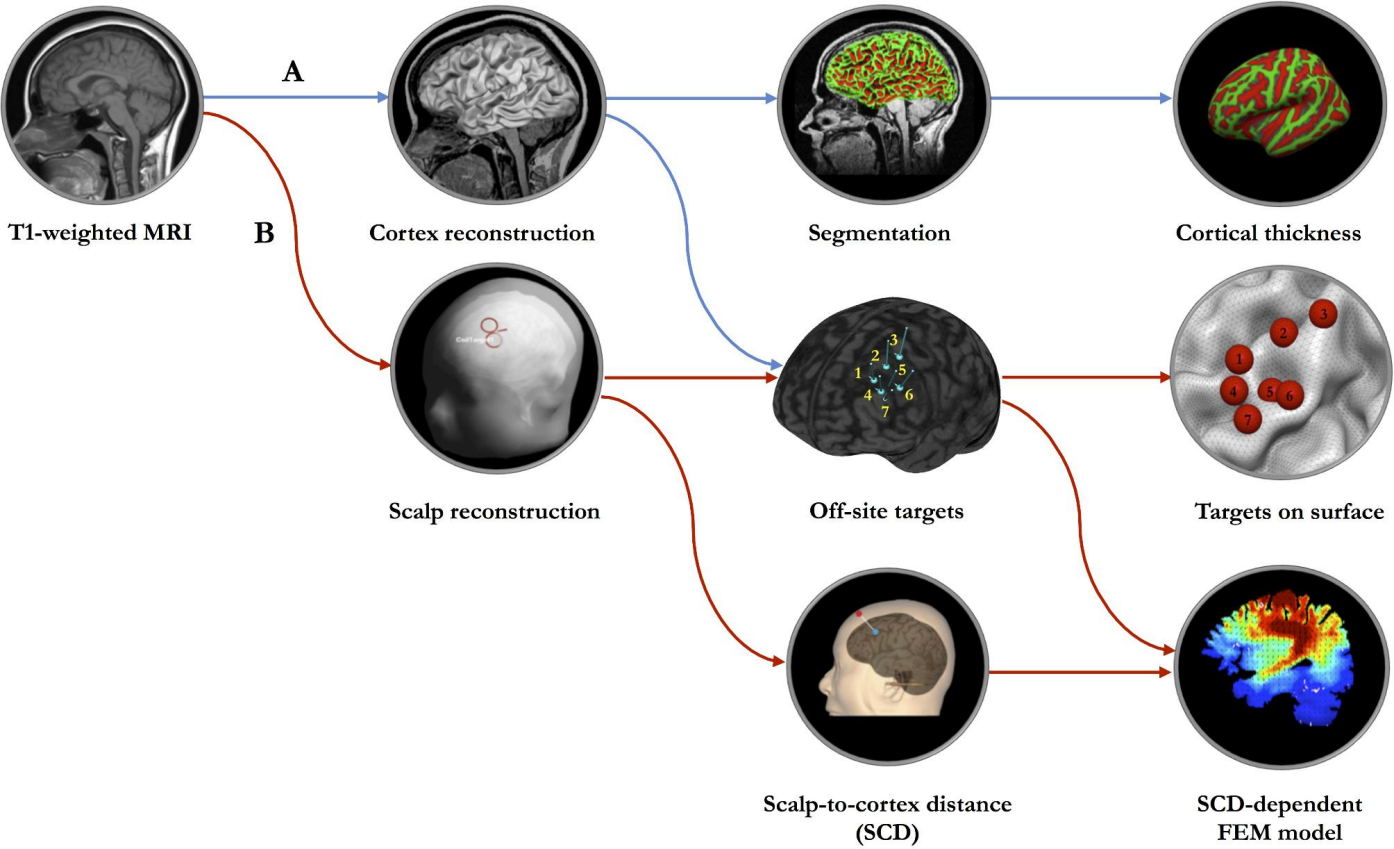
The framework of MRI-based brain morphometric analysis and computational model of SCD-dependent TMS-induced electric fields. (A) Cortex-based features. After cortex reconstruction and segmentation, the quantitative cortical measures of treatment target derived from BrainSuite contained cortical thickness and surface area. (B) Scalp-based features. After constructing the scalp and cortex, we localized the seven off-site treatment targets and their locations on cortical surface, measured the scalp-to-cortex distance (SCD) and constructed the head models of SCD-dependent electric fields using Finite Element Method (FEM).

Surface-based analysis of brain features, including cortical volume, surface area and cortical thickness, were performed by BrainSuite 19a (http://brainsuite.org/) (Fig. 1A). BrainSuite is an automatic cortical surface identification integrated package with the refined version of brain surface extraction (BSE), which is suitable for individuals with brain atrophy [19, 20]. To extract and quantify the region-specific morphometric features, we followed the standard BrainSuite pipeline with default parameters and employed the parcellation scheme on the basis of the Automated Anatomical Labeling (AAL) template.

Cortical thickness is calculated as an average of the distance from the white matter (WM) surface to the closest point on the pial surface and from that point back to the closest point to the WM surface. The measure of surface area is calculated using the triangular tessellation of the gray matter (GM) / WM interface (inner surface) and the WM / Cerebrospinal fluid (CSF) interface (pial surface) [21]. Cortical folding measured by gyrification index (GI) is a ratio of inner surface area to the area of an outer surface that smoothly encloses the cortex [22].

### SCD of left DLPFC

**Fig. 2:**
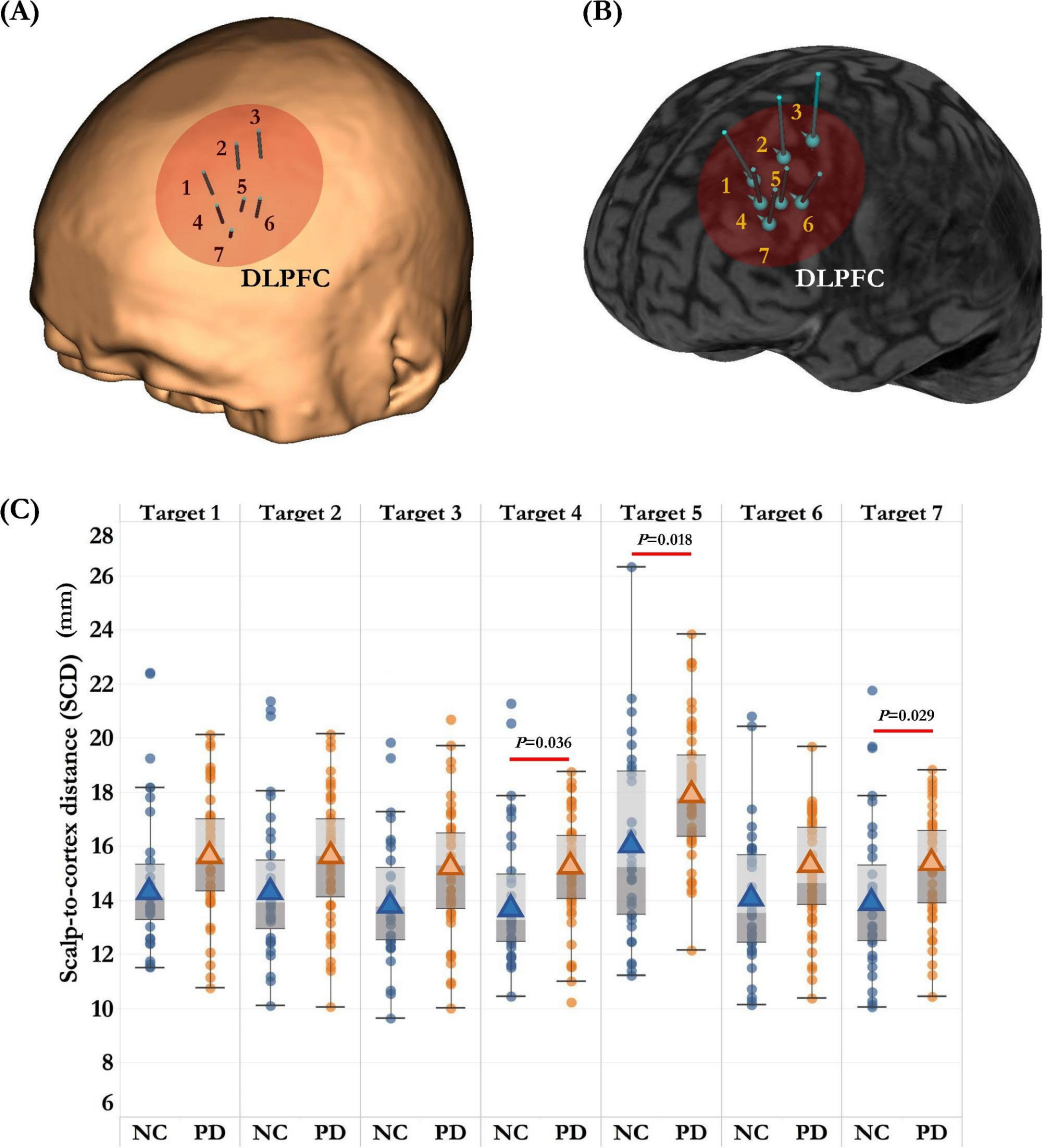
Comparisons of the scalp-to-cortex distance (SCD) in left dorsolateral prefrontal cortex (DLPFC) in early-stage PD patients and age-matched normal controls (NC). Data are displayed as mean ± SD. Seven targets of left DLPFC were located and marked on the scalp (A) and cortex (B), including: Brodmann Area (BA) 9 centre (Target 1); EEG F3 (Target 2); Average 5 cm (Target 3); Fitzgerald Target (Target 4); Paus Cho Target (Target 5); Rusjan Target (Target 6); BA46 centre (Target 7). (C) The early-stage PD patients showed increased SCDs than normal controls across the seven targets of left DLPFC. Significant between-group differences of SCDs were found in Target 4 (*P* = 0.036), Target 5 (*P* = 0.018) and Target 7 (*P* = 0.029)

The MNI coordinates of the seven targets of left DLPFC were drawn from published studies (Fig. 2) [23–25], including: (1) Brodmann Area (BA) 9 centre: *x* = − 36, *y* = 39, *z* = 43; (2) Electroencephalography (EEG) F3: *x* = − 37, *y* = 26, *z* = 49; (3) Average 5 cm: *x* = − 41, *y* = 16, *z* = 54; (4) Fitzgerald Target: *x* = − 46, *y* = 45, *z* = 38; (5) Paus Cho Target: *x* = − 40, *y* = 31, *z* = 34; (6) Rusjan Target: *x* = − 50, *y* = 30, *z* = 36; (7) BA46 centre: *x* = − 44, *y* = 40, *z* = 29.

Scalp-to-cortex distance (SCD), as a geometric feature, was measured in the Brainsight TMS neuronavigation system (http://thebrainx.com/landscape/) [26]. Based on the structural MRI scans, we first reconstructed the 3D curvilinear of scalp and cortex, and then adjusted the MRI-to-head co-registration using the anterior commissure - posterior commissure (AC-PC) line in the MNI space. After co-registration, the locations of the targets on cortex were identified and pinpointed with the MNI coordinates of the seven targets of left DLPFC (*x, y, z*). To better mimic the realistic TMS treatment, the corresponding locations of the seven targets of left DLPFC on the scalp were targeted in the neuronavigation system by pointing the cursor to the scalp and then adjusting the orientation of the TMS coil from the midline at 45**°**. Euclidean distance (*D*_*i*_) was used to measure the distance between the coordinates locating on the scalp (*x*_*s*_, *y*_*s*_, *z*_*s*_) and the cortex (*x*_*c*_, *y*_*c*_, *z*_*c*_) in the MNI space with the following formula [15, 27]:

$$D_i = \sqrt{{\left({x}_{s}-{x}_{c}\right)}^{2}+{\left({y}_{s}-{y}_{c}\right)}^{2}+{\left({z}_{s}-{z}_{c}\right)}^{2}}$$

### FEM model of SCD-dependent E-fields

To establish a realistic head model of TMS, we applied the Finite Element Method (FEM) [28, 29], a well-established approach for integrating different brain tissues and cortical surface features, thereby allowing it to account for the impact of PD-related cortical changes. SimNIBS, as a state-of-the-art platform for the simulation of transcranial brain stimulation (http://simnibs.de/), allows for the computational calculations of the TMS-induced E-fields [30]. As part of SimNIBS pipeline, FEM model distinguishes between scalp, skull, CSF, GM and WM, of which the assigned values of conductivities are σ_skin_ = 0.465 S/m, σ_skull_ = 0.01 S/m, σ_CSF_ = 1.654 S/m, σ_GM_ = 0.276 S/m, and σ_WM_ = 0.126 S/m [31].

The first step in constructing the realistic head model was to generate a conductor model of the head. In order to create the finite element mesh, we assigned each voxel in structural MRI scans to a specific tissue type (Fig. 1B). As a recommended option in SimNIBS, we selected headreco in combination with the Statistical Parametric Mapping (SPM12) toolbox (https://www.fil.ion.ucl.ac.uk/spm/software/spm12/) for achieving an accurate segmentations of brain tissues. The second step was to clean the tissue maps by applying morphological operations, and then use the tissue maps to create surface reconstructions. Finally, the FEM mesh was generated by filling in the tetrahedrons between the surfaces of tissue using Gmsh (http://gmsh.info/).

After constructing the realistic head model, the simulation of single-pulse TMS begin by adding the coil (Magstim 70 mm figure-8 coil) to the scalp. In this step, targets on the scalp were shifted to form the shape of the coil, while keeping good quality elements. Afterwards, the body of the coil was constructed by filling in tetrahedra. Simulations were run with a TMS pulse of 1.00 × 10e6 A/s, a Magstim 70 mm figure-8 coil over the targets of left DLPFC (i.e., F3 in International 10–20 system but modified with individual MNI coordinates on the cortex). Default conductivities of the toolbox were used for the different compartments as mentioned above [31].

### Quantitative measures of SCD-dependent E-fields

Considering the E-fields refer to a vector field, both intensity and focality of the simulated E-fields are quantified and visualized as norm or strength (i.e., vector length or magnitude) [31]. The E-fields magnitude of each target was quantified as the 95%, 99% and 99.9% of E-fields strength (Norm E) (Fig. 4A). To avoid the outlier effects, the peak value of E-fields magnitude (*E*_*max*_) is defined as the 99.9% of E-fields strength. The focality of E-fields is measured as the GM volume with the E-fields greater or equal to 50-75% of the peak value. TMS-affected cortical volume was quantified as the volume corresponding to the 50% (*Foc*_*50*_) and 75% (*Foc*_*75*_) of the maximum E-fields.

**Fig. 3:**
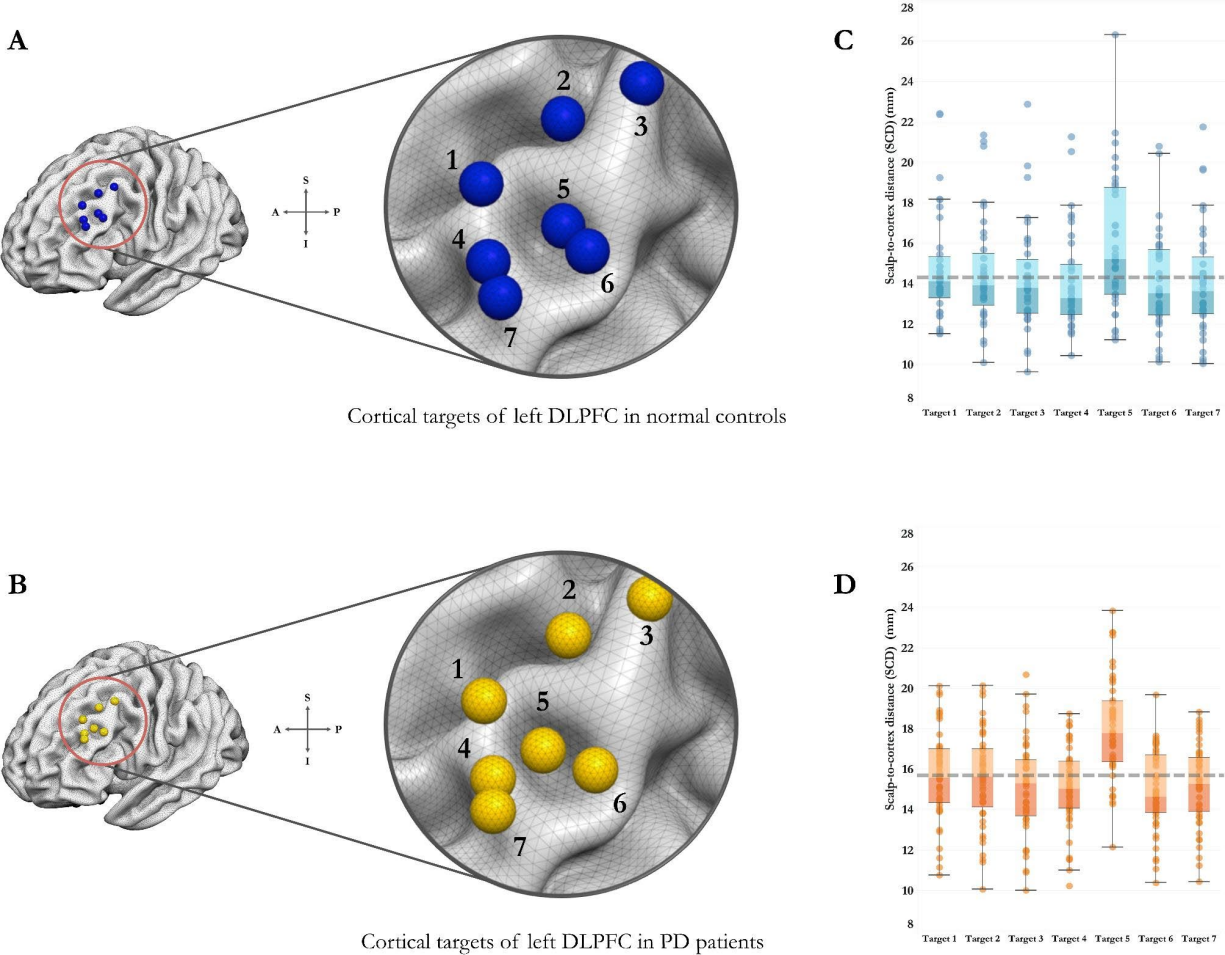
Illustrations of the target-specific scalp-to-cortex distance (SCD) and its variances in early-stage Parkinson’s disease (PD) patients and age-matched normal controls (NC). The spatial distributions of the seven treatment targets were demonstrated on the cortical area of left dorsolateral prefrontal cortex (DLPFC) in NC (A) and PD patients (B). Variances in the SCDs of the treatment targets of left DLPFC in PD patients (D) were higher than the SCDs in NC (C) (Grey dotted line represents the mean value of SCD across the seven targets). Data are displayed as mean ± SD.

**Fig. 4:**
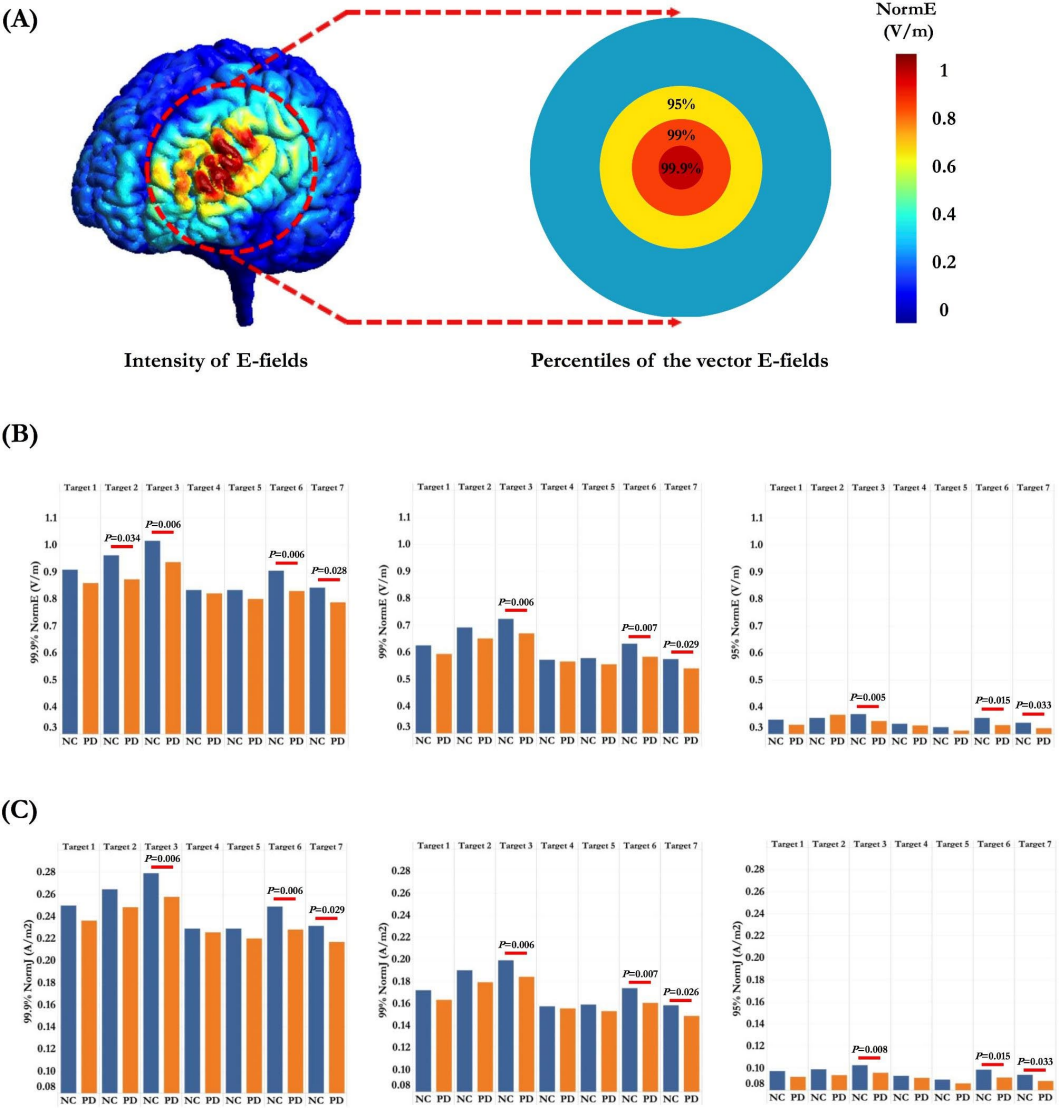
Comparisons of the magnitude and current intensity of the SCD-dependent TMS-induced E-fields in normal controls (NC) and Parkinson’s disease (PD) patients. Data are displayed as mean ± SD. (A) The magnitude of the E-fields was measured as the 95% (yellow), 99% (orange) and 99.9% (*E*_*max*_) (red) of the strength of simulated E-fields. The early-stage PD patients showed significant decreased *E*_*max*_ (V/m) (NormE) (B) and current intensity (NormJ) (A/m^2^) (C) in Target 3 (EEG F3), Target 6 (Rusjan Target) and Target 7 (BA46 centre). SCD, Scalp-to-cortex distance; TMS, Transcranial magnetic stimulation; E-fields, Electric fields

### Statistical analysis

The differences of demographics, clinical features, cognitive performance and the SCDs of left DLPFC were tested either with the chi-square (*χ*^2^) test for categorical variable or with independent two samples *t*-test for continuous variables. The groupwise comparisons of the morphometric features were conducted by using the code embedded in the MATLAB (http://neuroimage.usc.edu/neuro/Resources/BST_SVReg_Utilities). The corrections of multiple comparisons were performed by the above code using false discovery rate (FDR) estimation; 2-sided *p* < 0.05 is considered statistically significant. The quantitative measures of SCD-dependent E-fields were compared between the seven targets of left DLPFC using repeated measures analysis of variance (ANOVA), followed by post-hoc *t*-tests with Bonferroni correction. The receiving operating characteristic (ROC) analysis was conducted to evaluate the power of cognitive and brain measures in differentiating the individuals with different clinical statuses. The *χ*^2^ test, ANOVA, Pearson correlation coefficients and ROC analysis were performed using R (version 3.6.2) and R Studio (version 1.3.959) software.

## Results

### Demographics, cognitive function, and brain morphometry

Demographics in terms of age and sex, global cognition (measured by MMSE), global brain morphometry and the morphometric features of left DLPFC, including GM volume, WM volume, surface area, cortical thickness and folding (measured by gyrification index), were comparable between PD patients and normal controls (Table 1).

**Table 1:** Demographics, clinical symptoms and global morphometric features

	Normal controls (n = 36)	PD patients (n = 47)	*t* (*χ*^2^)	*p* value
Age (years)	66.03 ± 8.92	67.21 ± 8.61	-0.612	0.542
Sex (M/F)	16:20	19:28	0.895	0.611
MMSE	29.05 ± 1.32	28.75 ± 1.07	0.791	0.434
HY	-	1.91 ± 0.47	-	-
Global brain morphometry				
Mean CT (mm)	3.95 ± 0.21	3.98 ± 0.24	-0.751	0.455
Mean GMV (×10^3^ mm^3^)	6.49 ± 0.59	6.55 ± 0.68	-0.397	0.692
Mean WMV (×10^3^ mm^3^)	3.83 ± 0.54	3.91 ± 0.59	-0.591	0.556
Morphometry of left DLPFC				
CT (mm)	4.39 ± 0.42	4.38 ± 0.41	0.067	0.947
GMV (×10^3^ mm^3^)	13.39 ± 1.75	13.27 ± 1.99	0.301	0.764
WMV (×10^3^ mm^3^)	6.86 ± 1.37	7.05 ± 1.67	-0.564	0.574
Pial surface area (×10^3^ mm^2^)	5.53 ± 0.69	5.49 ± 0.94	0.261	0.796
Inner surface area (×10^3^ mm^2^)	3.28 ± 0.61	3.27 ± 0.68	0.092	0.927
Gyrification index	1.71 ± 0.22	1.70 ± 0.21	0.252	0.801

Note. Data are raw scores and presented as mean ± SD.

Abbreviations: PD = Parkinson’s disease; MMSE = The Mini-Mental State Exam; HY = Hoehn and Yahr Scale; CT = Cortical thickness; GMV = Gray matter volume; WMV = White matter volume

### Comparisons of the SCDs in left DLPFC

Compared to normal controls, PD patients had increased SCDs across the seven targets of left DLPFC (Table 2). As depicted in Fig. 2B, significant differences of SCDs were found in specific targets, including Fitzgerald Target (Target 4: *t* = -2.14, *p* = 0.036), Paus Cho Target (Target 5: *t* = -2.42, *p* = 0.018), and BA 46 centre (Target 7: *t* = -2.23, *p* = 0.029). Within each group, the SCD of Paus Cho Target was significantly greater than the other targets (Normal controls: *p* = 0.005; PD patients: *p* < 0.001). To better understand the heterogeneity in SCD, we further calculated the variance of the SCDs of the seven targets of left DLPFC using the formula: ${S}^{2}= \frac{\sum {(x}_{target}-{x}_{mean}){ }^{2}}{N-1}$, *x*_*target*_ represents the SCD of each target, *x*_*mean*_ represents the average value of SCD across the seven targets, *N* represents the number of targets. Compared to normal controls, PD patients showed higher variability in SCDs between the seven targets of left DLPFC (*S*^*2*^: Normal controls: 2.266 ± 1.623, PD patients: 3.472 ± 2.458, *t* = -2.152, *p* = 0.034) (Fig. 3).

**Table 2:** Scalp-to-cortex distance (SCD) of the seven targets of left DLPFC

SCD	Normal controls (n = 36)	PD patients (n = 47)	*t* (*χ*^2^)	*p* value
1. BA9 center	14.75 ± 2.68	15.71 ± 2.26	-1.78	0.079
2. EEG F3	14.53 ± 2.67	15.53 ± 2.32	-1.83	0.072
3. Average 5 cm	14.24 ± 2.64	15.08 ± 2.31	-1.55	0.125
4. Fitzgerald Target	14.04 ± 2.49	15.11 ± 2.02	**-2.14**	**0.036**
5. Paus Cho Target	16.28 ± 3.98	18.02 ± 2.52	**-2.42**	**0.018**
6. Rusjan Target	14.03 ± 2.49	14.94 ± 2.02	-1.85	0.069
7. BA 46 center	14.06 ± 2.76	15.22 ± 1.99	**-2.23**	**0.029**

Note. Data are raw scores and presented as mean ± SD.

Abbreviations: DLPFC = Dorsolateral Prefrontal Cortex; PD = Parkinson’s Disease; BA = Brodmann Area; EEG = Electroencephalography.

### Quantitative measures of SCD-dependent E-fields

SCD-dependent E-fields intensity and focality were simulated and quantified for the seven targets of left DLPFC, indicating a heterogeneous pattern of E-fields distribution across the targets (Supplementary Fig. 1). The average *E*_*max*_ in PD patients was 0.858 V/m, which was weaker than the value in normal controls (0.908 V/m). Significant differences of the magnitude and current density of the SCD-dependent E-fields were found in specific targets, including EEG F3 (Target 2: *t* = 2.129, *p* = 0.041), Average 5 cm (Target 3: *t* = 2.919, *p* = 0.006), Rusjan Target (Target 6: *t* = 2.936, *p* = 0.006), BA 46 centre (Target 7: *t* = 2.298, *p* = 0.028) (Fig. 4) (Supplementary Tables 1 and Table 2). The average *Foc*_*75*_ was 5.59 cm^3^ in PD patients and 5.53 cm^3^ in normal controls. The *Foc*_*75*_ of the SCD-dependent E-fields varied between 5.16 cm^3^ and 6.31 cm^3^ (Supplementary Table 3), which corresponded to 38.5-46.5% of the GM volume in left DLPFC in normal controls and 39.4-47.8% of the GM volume in left DLPFC in PD patients. Among the seven targets of left DLPFC, the peak values were significantly decreased in Rusjan Target (Target 6: *t* = -3.151, *p* = 0.003) and BA 46 center (Target 7: *t* = -2.319, *p* = 0.027) in PD patients.

As to the heterogeneity in TMS-induced E-fields focality, we calculated the variance of the SCD-dependent E-fields using the same formula: ${S}^{2}= \frac{\sum {(x}_{target}-{x}_{mean}){ }^{2}}{N-1}$, *x*_*target*_ represents the target-specific E-fields, *x*_*mean*_ represents the average value of E-fields across the seven targets, *N* represents the number of targets. Compared to normal controls, PD patients showed comparable variance in the *Foc*_*75*_ of the SCD-dependent E-fields (Normal controls: 0.2 ± 0.03, PD patients: 0.21 ± 0.02, *t* = -0.297, *p* = 0.769), indicating that PD patients had heterogeneous distribution of the E-fields in TMS-affected cortical volume (Supplementary Fig. 2).

### Associations between SCD and clinical features

Using age and sex as covariates, the relationship between SCD, the magnitude and focality of the E-fields and morphometric features was examined at each target of left DLPFC. We found that the gyrification index of left DLPFC was significantly correlated with the target-specific SCD in PD patients, including the Paus Cho Target (Target 5: *r* = 0.384, *p* = 0.008), Rusjan Target (Target 6: *r* = 0.348, *p* = 0.017) and BA 46 centre (Target 7: *r* = 0.356, *p* = 0.014), but not in normal controls.

### ROC analysis

To classify the individuals with PD, the value of the area under the ROC curve (AUC) was used to test the discriminant power of the brain and clinical features. We found that neither MMSE nor other brain measures showed a significant discriminative power (Supplementary Fig. 3); while the geometric measure of the mean SCD of left DLPFC, had a better performance to differentiate PD patients from normal controls (AUC = 0.733, *p* = 0.012). Moreover, all the SCDs of left DLPFC targets showed significant power to discriminate PD patients.

## Discussion

TMS is a non-invasive brain stimulation technology that is being increasingly employed as a non-pharmacological treatment for age-related neurodegenerative diseases. The variety of treatment targets and the concomitant E-fields-dependent dosimetry limits the personalized applications of TMS in clinical practice. In this study, we examined and quantified the scalp-to-cortex distance (SCD) of seven commonly used targets of left DLPFC and its impact on the intensity and focality of the simulated E-fields through computational realistic head models in early-stage PD patients.

### Heterogeneity in treatment targets

With the rapid advances in imaging and analytical tools, the stimulation-targeting rules have been switched from scalp-based to cortex / laminar-specific targeting [32, 33]. In recent years, non-invasive neuroimaging technologies, such as MRI, functional near-infrared spectroscopy (fNIRS) and positron emission tomography (PET), offer great promise for determining the TMS treatment targets at individual level. Using functional MRI as an example, functional connectivity-based DLPFC targeting for the treatment of major depressive disorders has been well developed and evaluated [23, 34, 35]. Although there were significant differences in target-based functional connectivity among DLPFC targets [25, 36, 37], the heterogeneity in targeting highlights the necessity and importance of pinpointing the optimal stimulation sites within the DLPFC rather than a general anatomical area. Despite these informative findings, a pertinent question is concerned with the more efficient strategy for determining the precise targets for TMS treatment or, alternatively, whether optimal targeting within a TMS target is a quick and plausible way for clinical populations, particularly those with neurodegenerative diseases.

Based on the published TMS studies in PD patients, a figure-8 coil was positioned over the left DLPFC through scalp-based targeting, including Average 5 cm [38, 39], EEG F3 [11], or neuronavigated targeting with the MNI coordinates close to BA46 centre [10, 12]. However, when comes to senior adults or patients with dementia, there are several issues that need to be addressed in considering the TMS treatment targets. First, it is critical to specifically map the cortical site for stimulation: whether this is a set of predefined MNI coordinates within the DLPFC, or a specific surface on the cortex. Second to this is the approach to ensure the TMS coil is positioned over the corresponding scalp site with right orientation over the stimulation site of left DLPFC. Third, it is critical to localize the cortical target in combination with the surface features of DLPFC.

The surface features of cortical targets vary from region to region in the individuals with different cognitive statuses. Unlike AD and Frontotemporal dementia (FTD), PD is not a typical age-related neurodegenerative disease with cortical atrophy [40, 41]. In cognitively normal early-stage PD patients, there was little or no cortical atrophy or thinning as compared to normal controls. Indeed, the results of global morphometry and the morphometric features of left DLPFC in PD patients accord with the documented imaging studies [41, 42]. Although PD patients had comparable brain volumes and cortical thickness, the SCD of left DLPFC in PD patients were generally greater than in normal controls. It should be noted that the distance between the TMS coil (i.e., scalp) to the cortex is a key technical parameter that determines the motor-evoked potential (MEP) and the output of TMS (i.e., region-specific dosage) [43–45]. Furthermore, even though TMS coils vary in type and size, the range of TMS penetration depth is around 0.9–3.5 cm [46], figure-8 coil has a penetration depth of 1.5 to 2.5 cm [47]. Six of the seven targets within left DLPFC had SCDs greater than 1.5 cm. Of note, the Paus Cho Target (Target 5) located at the sulcal crown with a greatest SCD had the lower intensity and focality of the TMS-induced E-fields than the other targets. Interestingly, in the context of global increased SCD, the heterogeneity in the SCDs of the targets within left DLPFC was also significantly increased in early-stage PD patients, which is thought to be a preferentially affected neurophenotype depending upon neurodegeneration and may have potential clinical utilities.

### SCD as new marker for PD patients

At individual level, although the predefined MNI coordinates and the labeled SCD-adjusted targets on the cortex were located within the surface of left DLPFC, the variations in the SCDs across the targets indicate greater intra-individual variability in geometric features and the corresponding E-fields focality in PD patients. At the group level, increased SCDs, rather than global cognition and morphometric features, demonstrated strong positive correlations with the cortical folding of treatment target, as well as a significant power to differentiate early-stage PD patients from age- / morphometry-matched normal controls, implying that the vector-like SCD, as a key technical parameter, is more than simply useful for guiding TMS coil (i.e., scalp site) to the precise target located on the highly folded cortex. Of note, the impacts of morphometric features on the simulated E-Fields, particularly cortical folding, have been tested in the studies of TMS [48], and other modalities of transcranial brain stimulation [49]. Therefore, this intrinsically connects geometric and morphometric features (i.e., SCD and cortical folding) with precise identification of the stimulation targets, which makes it possible for optimizing the process of TMS targeting in combination with the underlying surface morphometry.

Our findings show that the targets close to the gyral crown of the left DLPFC, such as Average 5 cm, EEG F3, BA9 centre, and Rusjan Target, had stronger E-fields intensity than the other targets, which are consistent with previous findings [50, 51]. Meanwhile, we found that the SCDs of the inferior targets of left DLPFC, such as Paus Cho Target, Rusjan Target and BA 46 centre, are significantly associated with the higher degree of cortical folding, but not other morphometric features. It should be noted that BA9 and BA46 are well-established DLPFC subregions with diverse cytoarchitecture and neural circuits among the seven targets [52]. For instance, BA9 and BA46 are disproportionately affected in brain disorders, with BA46 showing the most predominant layer II thinning, but not BA9 [50]. In our results, the distinct patterns of SCD-dependent intensity and focality of the E-fields in early-stage PD patients highlighted the feasibility, necessity and importance of targeting the left DLPFC subregions through realistic geometric modeling for the first time. As a result, for transcranial brain stimulation, region-specific surface-based brain features provide valuable information of the cortical landscape underneath TMS coil, which is expected to combine morphometric and geometric features in the construction of personalized head model.

Our findings might pave the way for personalized transcranial brain stimulation that targets the disease-specific optimal targets using geometric feature-informed head models. At a minimum, our study supports a quick and plausible assessment of SCD for localizing the treatment target sites on the scalp and cortex for senior adults and PD patients. This may enable disease-specific selection of stimulation sites and TMS dosage adjustment across different brain regions. In prior research, we found region-specific SCD and its impacts on E-fields in normal ageing people, as well as patients with mild cognitive impairment (MCI) and AD [16, 27]. The current work may potentially be useful in developing an advanced, next-generation therapeutic framework of personalized brain stimulation for patients with neurodegenerative diseases. The SCD and SCD-dependent E-fields could be partitioned into a pre-treatment parameter for optimizing stimulation dose in computational E-fields modeling. Based on the findings, we recommend that the treatment targets located on gyral crown with a shorter SCD have a higher intensity and focality of E-fields than the other targets for modulating or enhancing motor function, cognitive functions, sleep quality and mood in early-stage PD patients.

### Limitations and future directions

The current work illustrates the heterogeneity in region-specific SCD and the corresponding stimulation-induced electric fields intensity in early-stage PD patients, however the findings should be interpreted with respect to several limitations. First, this study examined the brain features in early-stage PD patients and normal controls from two separate datasets. Although the inclusion and exclusion criteria for PD patients were fairly standard, and the brain features of PD patients in two datasets were comparable, the cognitive status might have influenced the results. Besides, because the sample number is moderately low, a bigger dataset for validation is necessary before applying to clinical practice. Second, because the current findings are based on cross-sectional data, they cannot be extrapolated to ageing effects or disease progression. Furthermore, several key variables linked to metabolism and genetic risk factors were not provided in the NEUROCON and Tao Wu datasets, limiting the capacity to examine the genetic determinants of the brain (i.e., neurogenetics).

Future research should validate the current results in a bigger PD dataset with genetic information, such as Parkinson’s Progression Markers Initiative (PPMI) and examine the heterogeneity of geometric and morphometric brain measures in different age groups and other main types of neurodegenerative diseases (e.g., AD, frontotemporal dementia). In terms of technique, it is critical to note that the scalp-to-cortex distance and gyrification may influence the stimulation dose at individual level. For addressing this issue, future work will in-depth investigate the geometric features of the treatment targets and plug them in the optimization of coil placement and parameter estimations in transcranial brain stimulation studies. Last but not least, the MRI-informed head model should be validated and used in other advanced modality of brain stimulation, such as transcranial ultrasound stimulation (TUS) [53, 54].

## Conclusion

In conclusion, the current work adds to the growing knowledge that individual brain features can aid in precisely localizing treatment targets and optimizing the dose for transcranial brain stimulation. An integrated imaging-informed computational model allows brain measures and electric fields matrices to simultaneously inform one another, resulting in a more radiomic and realistic understanding of optimal TMS targeting in senior adults and the individuals suffering from Parkinson’s disease. The region-specific brain features identified here provide a new direction for dissecting the heterogeneity, complementing previous attempts to develop personalized treatment strategies.

## Electronic supplementary material

Below is the link to the electronic supplementary material.

**Additional file 1: Supplementary Table 1**. The magnitude and distribution of SCD-dependent electric fields in PD patients and normal controls. **Supplementary Table 2**. The distribution of SCD-dependent electric fields in PD patients and normal controls. **Supplementary Table 3**. The focality of SCD-dependent electric fields in PD patients and normal controls. **Supplementary Figure 1**. Head models of transcranial magnetic stimulation (TMS)-induced SCD-dependent electric fields (E-fields) in early-stage PD patients. **Supplementary Figure 2**. Comparisons of the focality of the SCD-dependent transcranial magnetic stimulation (TMS)-induced electric fields (E-fields) in normal controls (NCs) and early-stage Parkinson?s disease (PD) patients. **Supplementary Figure 3**. Receiver-operator characteristic (ROC) curves for the cognition and the geometric and morphometric features with differential values in early-stage Parkinson?s disease (PD) patients.

## Data Availability

The datasets used and analyzed during the current study are available from the corresponding author on reasonable request.

## Abbreviations

AAL: Automated Anatomical Labeling
AD: Alzheimer’s disease
BA: Brodmann area
DLPFC: Dorsolateral prefrontal cortex
EEG: Electroencephalography
E-fields: Electric fields
FEM: Finite Element Method
FTD: Frontotemporal dementia
GM: Gray matter
HY: Hoehn and Yahr Scale
MCI: Mild cognitive impairment
MEP: Motor-evoked potential
MMSE: Mini-mental state examination
MNI: Montreal Neurological Institute
MPRAGE: Magnetization-prepared rapid gradient-echo
MRI: Magnetic resonance imaging
PD: Parkinson’s disease
PFC: Prefrontal cortex
SCD: Scalp-to-cortex distance
ROC: Receiving operating characteristic
TMS: Transcranial magnetic stimulation
TUS: Transcranial ultrasound stimulation
WM: White matter
