# Supplementary material for: Pinpointing the precise stimulation targets for brain rehabilitation in early-stage Parkinson’s disease
Source: BMC Neurosci. 2023 Mar 29;24:24. doi: 10.1186/s12868-023-00791-7 (PMC10061909; doi:10.1186/s12868-023-00791-7)

Supplementary Table 1. The magnitude and distribution of SCD-dependent electric fields in PD patients and normal controls

| **Targets of left DLPFC** | 99.9% electric fields (V/m) | | | | 99% electric fields (V/m) | | | | 95% electric fields (V/m) | | | |
| --- | --- | --- | --- | --- | --- | --- | --- | --- | --- | --- | --- | --- |
|  | NC | PD | *t* value | *p* value | NC | PD | *t* value | *p* value | NC | PD | *t* value | *p* value |
| Target 1 | 0.91±0.09 | 0.86±0.07 | 1.787 | 0.083 | 0.63±0.06 | 0.59±0.05 | 1.791 | 0.082 | 0.35±0.03 | 0.34±0.03 | 1.869 | 0.071 |
| Target 2 | 0.96±0.09 | 0.87±0.14 | **2.129** | **0.041** | 0.69±0.07 | 0.65±0.05 | 1.974 | 0.057 | 0.36±0.03 | 0.37±0.15 | -0.321 | 0.751 |
| Target 3 | 1.02±0.09 | 0.94±0.07 | **2.919** | **0.006** | 0.72±0.06 | 0.67±0.05 | **2.913** | **0.006** | 0.37±0.03 | 0.35±0.02 | **2.971** | **0.005** |
| Target 4 | 0.83±0.18 | 0.82±0.06 | 0.313 | 0.756 | 0.57±0.12 | 0.56±0.04 | 0.265 | 0.793 | 0.34±0.07 | 0.33±0.03 | 0.383 | 0.704 |
| Target 5 | 0.83±0.11 | 0.79±0.06 | 1.146 | 0.261 | 0.58±0.07 | 0.56±0.04 | 1.177 | 0.247 | 0.33±0.04 | 0.31±0.02 | 1.249 | 0.221 |
| Target 6 | 0.91±0.09 | 0.83±0.06 | **2.936** | **0.006** | 0.63±0.06 | 0.58±0.04 | **2.877** | **0.007** | 0.36±0.04 | 0.33±0.02 | **2.571** | **0.015** |
| Target 7 | 0.84±0.08 | 0.79±0.06 | **2.298** | **0.028** | 0.58±0.05 | 0.54±0.04 | **2.282** | **0.029** | 0.34±0.03 | 0.32±0.02 | **2.222** | **0.033** |

Note. Data are raw scores and presented as mean ± SD.

Abbreviations: SCD=Scalp-to-cortex distance; DLPFC=Dorsolateral prefrontal cortex; PD=Parkinson’s disease; NC=Normal controls.

Supplementary Table 2. The distribution of SCD-dependent electric fields in PD patients and normal controls

| **Targets of left DLPFC** | 99.9% electric fields (A/m^2^) | | | | 99% electric fields (A/m^2^) | | | | 95% electric fields (A/m^2^) | | | |
| --- | --- | --- | --- | --- | --- | --- | --- | --- | --- | --- | --- | --- |
|  | NC | PD | *t* value | *p* value | NC | PD | *t* value | *p* value | NC | PD | *t* value | *p* value |
| Target 1 | 0.25±0.03 | 0.24±0.02 | 1.792 | 0.082 | 0.17±0.02 | 0.16±0.01 | 1.779 | 0.084 | 0.09±0.01 | 0.08±0.01 | 1.889 | 0.067 |
| Target 2 | 0.26±0.03 | 0.25±0.02 | 1.976 | 0.056 | 0.19±0.02 | 0.18±0.01 | 1.968 | 0.057 | 0.10±0.01 | 0.09±0.01 | 2.019 | 0.051 |
| Target 3 | 0.28±0.02 | 0.26±0.02 | **2.906** | **0.006** | 0.20±0.02 | 0.18±0.01 | **2.936** | **0.005** | 0.11±0.01 | 0.09±0.01 | **2.903** | **0.006** |
| Target 4 | 0.23±0.05 | 0.23±0.02 | 0.317 | 0.753 | 0.16±0.03 | 0.16±0.01 | 0.273 | 0.786 | 0.09±0.02 | 0.09±0.01 | 0.391 | 0.699 |
| Target 5 | 0.23±0.03 | 0.22±0.02 | 1.143 | 0.261 | 0.16±0.02 | 0.15±0.01 | 1.183 | 0.245 | 0.09±0.01 | 0.08±0.01 | 1.246 | 0.221 |
| Target 6 | 0.25±0.03 | 0.23±0.02 | **2.934** | **0.005** | 0.17±0.02 | 0.16±0.01 | **2.905** | **0.006** | 0.10±0.01 | 0.09±0.01 | **2.562** | **0.015** |
| Target 7 | 0.23±0.02 | 0.22±0.02 | **2.277** | **0.029** | 0.16±0.01 | 0.15±0.01 | **2.332** | **0.026** | 0.09±0.01 | 0.08±0.01 | **2.229** | **0.033** |

Note. Data are raw scores and presented as mean ± SD.

Abbreviations: SCD=Scalp-to-cortex distance; DLPFC=Dorsolateral prefrontal cortex; PD=Parkinson’s disease; NC=Normal controls.

Supplementary Table 3. The focality of SCD-dependent electric fields in PD patients and normal controls

| **Targets of left DLPFC** | 75% E-fields focality (cm^3^) | | | | 50% E-fields focality (cm^3^) | | | |
| --- | --- | --- | --- | --- | --- | --- | --- | --- |
|  | NC | PD | *t* value | *p* value | NC | PD | *t* value | *p* value |
| Target 1 | 5.27±0.09 | 5.32±0.08 | -1.728 | 0.093 | 19.01±0.47 | 19.19±0.36 | -1.402 | 0.171 |
| Target 2 | 6.22±0.15 | 6.31±0.13 | -1.954 | 0.059 | 20.62±0.51 | 20.89±0.51 | -1.641 | 0.111 |
| Target 3 | 6.06±0.16 | 6.09±0.13 | -0.578 | 0.567 | 21.21±0.51 | 21.54±0.43 | **-2.095** | **0.044** |
| Target 4 | 5.27±0.27 | 5.25±0.06 | 0.328 | 0.745 | 20.52±1.86 | 20.15±0.37 | 0.871 | 0.391 |
| Target 5 | 5.26±0.19 | 5.29±0.11 | -0.671 | 0.507 | 19.69±0.61 | 19.73±0.37 | -0.229 | 0.821 |
| Target 6 | 5.49±0.15 | 5.65±0.14 | **-3.151** | **0.003** | 20.71±0.57 | 21.34±0.87 | **-2.476** | **0.018** |
| Target 7 | 5.16±0.11 | 5.24±0.08 | **-2.319** | **0.027** | 20.23±0.46 | 20.52±0.47 | -1.804 | 0.081 |

Note. Data are raw scores and presented as mean ± SD.

Abbreviations: SCD=Scalp-to-cortex distance; DLPFC=Dorsolateral prefrontal cortex; PD=Parkinson’s disease; NC=Normal controls.

Supplementary Figure 1. Head models of transcranial magnetic stimulation (TMS)-induced SCD-dependent electric fields (E-fields) in early-stage PD patients. The first step was to localize the targets of left DLPFC with MNI coordinates (A). The second step was to place the Magstim 70-mm figure-of-8 coil with the adjustment of angle and orientation of TMS coil (B) and then run the simulation in SimNIBS (C).


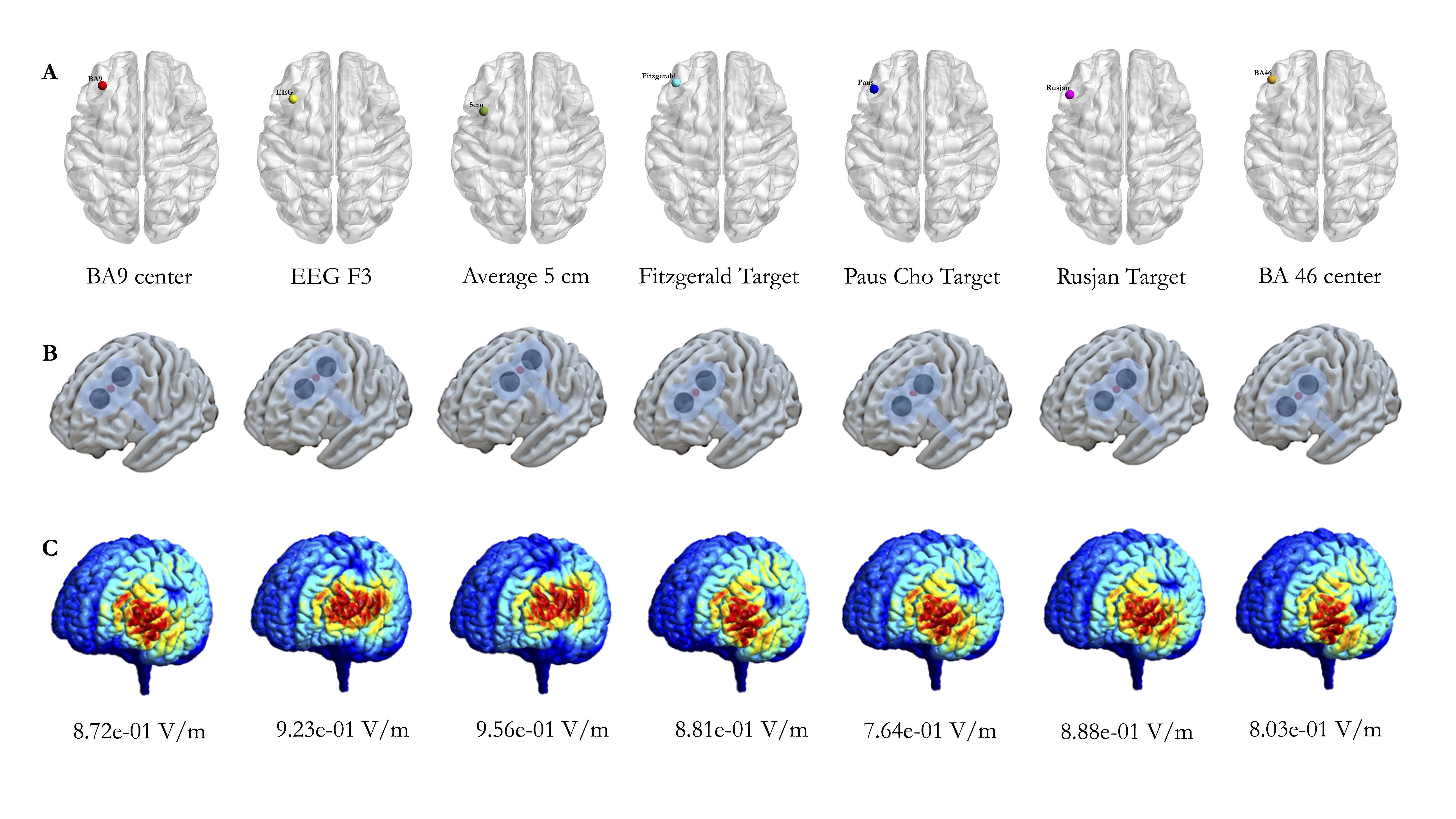


Supplementary Figure 2. Comparisons of the focality of the SCD-dependent transcranial magnetic stimulation (TMS)-induced electric fields (E-fields) in normal controls (NCs) and early-stage Parkinson’s disease (PD) patients. (A) TMS-affected cortical volume was quantified as the volume corresponding to the 50% (Foc50) (yellow) and 75% (Foc75) (orange) of the maximum E-fields. (B) The early-stage PD patients showed significant decreased Foc75 in Rusjan Target (Target 6) and BA46 centre (Target 7), and decreased Foc50 in EEG F3 and BA46 centre (Target 7) (C).


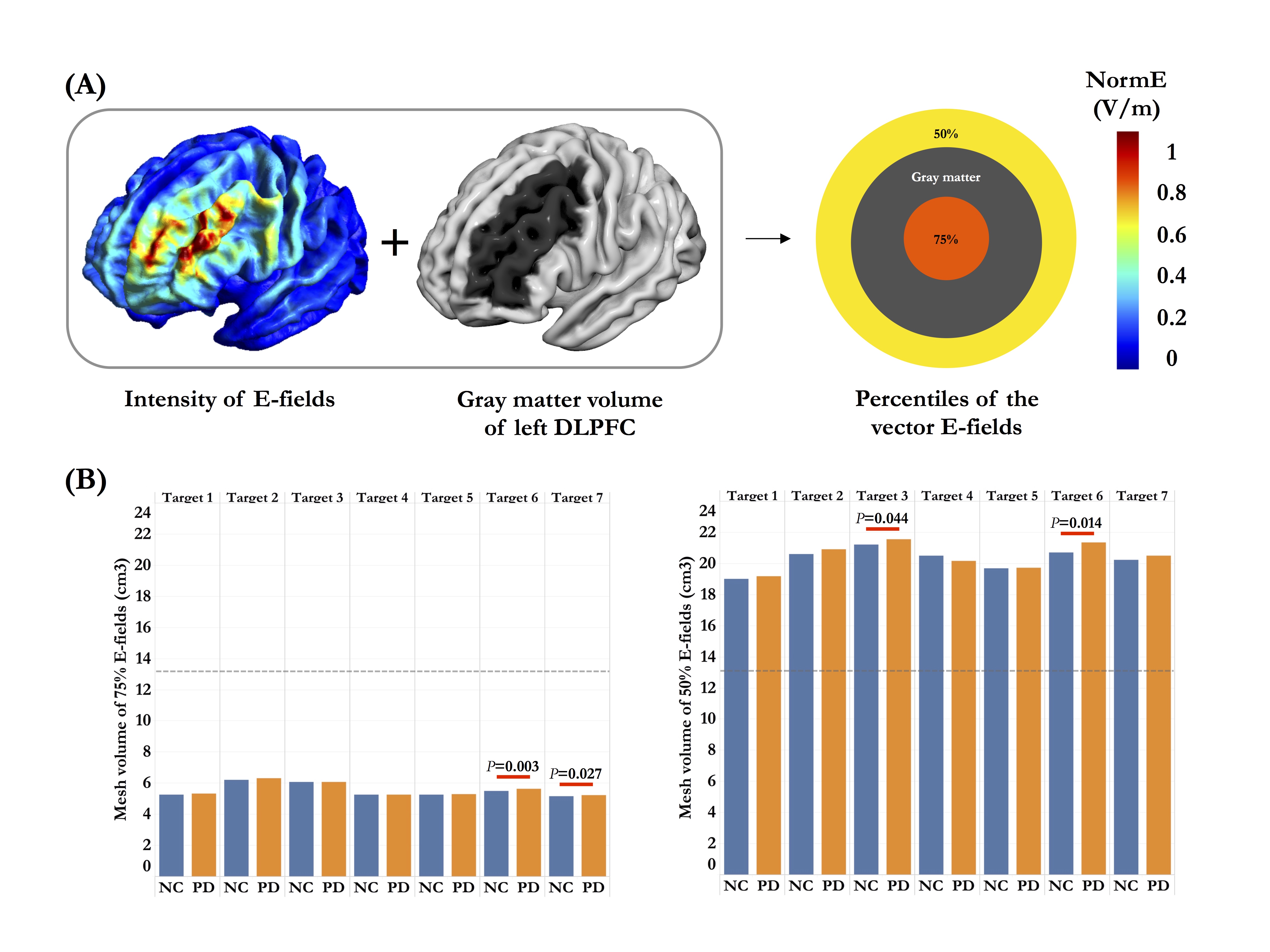


Supplementary Figure 3. Receiver-operator characteristic (ROC) curves for the cognition and the geometric and morphometric features with differential values in early-stage Parkinson’s disease (PD) patients. (A) Among all the measures, the mean SCD (A) and the SCDs of the targets of left DLPFC (B) showed a significant discriminative value in early-stage PD patients.


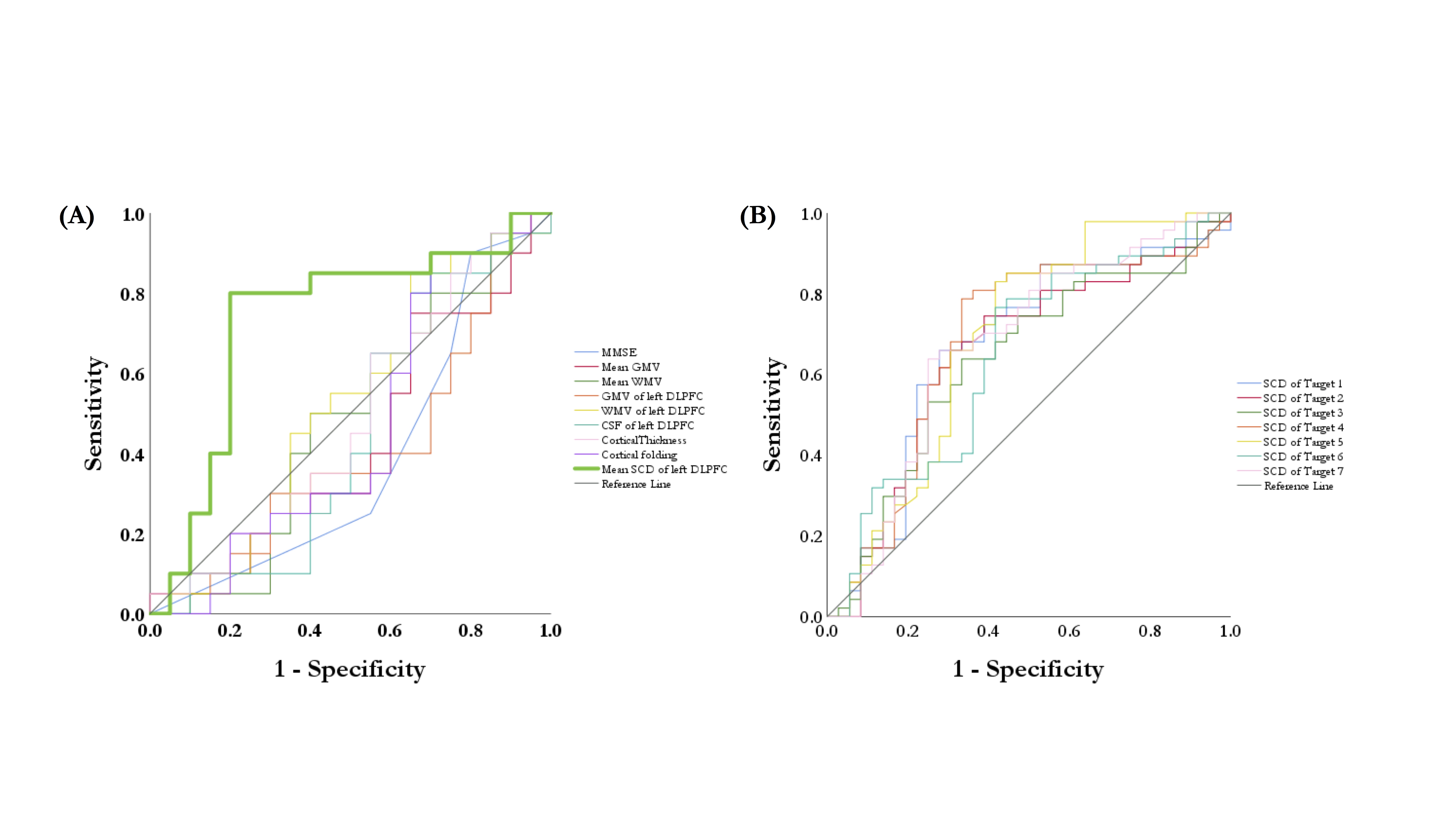

Supplement: Supplementary file 1 — Additional file 1: Supplementary Table 1. The magnitude and distribution of SCD-dependent electric fields in PD patients and normal controls. Supplementary Table 2. The distribution of SCD-dependent electric fields in PD patients and normal controls. Supplementary Table 3. The focality of SCD-dependent electric fields in PD patients and normal controls. Supplementary Figure 1. Head models of transcranial magnetic stimulation (TMS)-induced SCD-dependent electric fields (E-fields) in early-stage PD patients. Supplementary Figure 2. Comparisons of the focality of the SCD-dependent transcranial magnetic stimulation (TMS)-induced electric fields (E-fields) in normal controls (NCs) and early-stage Parkinson?s disease (PD) patients. Supplementary Figure 3. Receiver-operator characteristic (ROC) curves for the cognition and the geometric and morphometric features with differential values in early-stage Parkinson?s disease (PD) patients. [file 12868_2023_791_MOESM1_ESM.docx]
